# Hyperthermic Intraperitoneal Chemotherapy Versus Systemic Chemotherapy in Recurrent Platinum-Sensitive Ovarian Cancer NCI Case Control Study

**DOI:** 10.31557/APJCP.2019.20.2.621

**Published:** 2019

**Authors:** Gamal Amira, Ahmed Morsi, Ihab Samy Fayek, Osman Mansour, Heba Nader

**Affiliations:** 1 *Department of Surgical Oncology, *; 2 *Department of Medical Oncology, National Cancer Institute, Cairo University, Egypt.*

**Keywords:** Hyperthermic intraperitoneal chemotherapy, recurrent platinum, sensitive ovarian cancer

## Abstract

**Objectives:**

To assess the efficacy of cytoreductive surgery (CRS) combined with hyperthermic intraperitoneal chemotherapy (HIPEC) in recurrent platinum-sensitive ovarian cancer patients in comparison with standard intravenous chemotherapy in terms of progression free survival and overall survival.

**Methods:**

Retrospective case control study matching 15 cases with 20 controls with at least 24 months of follow up.

**Results:**

The two groups were comparable and well matched in all aspects. Median follow up was 36 months in cases and 38 months in controls. The PFS2 revealed a median of 6 months (range 2-14) in cases and 5 months (range 2-18) in controls. The median OS was 36 and 38 months in cases and controls respectively. No statistically significant difference between the cases and controls were observed in progression free survival (PFS2) and overall survival OS (P-value, 0.350 and 0.711 respectively). However, the PFS2 was in favor of cases and OS was in favor of controls without reaching significance. The percentage of patients who survived 5 years or more was 20% in cases and 35% in controls. The only issue in favor of HIPEC is the significant reduction in chemotherapeutic toxicity when given by the intraperitoneal way (P- value 0.003).

**Conclusion:**

According to our study, CRS and HIPEC do not seem to have impact on OS and PFS in the setting of recurrent platinum sensitive ovarian cancer. However, we recommend on going researches with much more refined selection criteria and with larger sample size.

## Introduction

Ovarian cancer is considered as the fourth highest cause of death in women. Ovarian cancer in the United states is still the first cause of gynecologic cancer deaths (Siegel et al., 2015). Despite maximum efforts have been made to develop a new way of screening, diagnosis, and treatment strategies, but the incidence and mortality of ovarian cancer did not change significantly (Pradjatma, 2016), and even after optimal cytoreduction followed by platinum-taxol based chemotherapy about 60% to 70% of patients develop a recurrence (Leitao and Chi, 2009).

Retrospective studies of platinum-based second-line therapies have identified two subgroups of patients with recurrent ovarian cancer: those with platinum-resistant disease and those with platinum-sensitive disease (Markman et al., 1991); the latter is defined by a relapse-free period of more than 6 months following a response to the final dose of platinum treatment.

Patients with platinum-sensitive disease are retreated with a platinum or platinum-containing combination, such as carboplatin (Pfisterer et al., 2006; Wagner et al., 2012; Pitakkarnkul et al., 2013). However, recurrence is still considered incurable. For this reason, researches trying novel treatments are worthy considered and should be compared to the current treatments.

Many studies have been conducted to test the efficacy of cytoreductive surgery (CRS) and hyperthermic intraperitoneal chemotherapy (HIPEC) in patients with recurrent ovarian cancer especially in that subgroup of patients with platinum sensitive disease. These trials showed promising results in terms of PFS and OS (Skaznik-Wikiel et al., 2012; Spiliotis et al., 2015; Deraco et al., 2012; Faggotti et al., 2012). This study represents our experience of CRS and HIPEC in the NCI, Cairo University, concerning this subset of patients with peritoneal carcinomatosis resulting from recurrent platinum sensitive ovarian cancer, since the introduction of the practice of HIPEC in the NCI for treating peritoneal surface malignancy.

## Materials and Methods

*Patients and Methods*

The study protocol was approved by the ethics committee of the National Cancer Institute, Cairo University. Informed consent was obtained from all patients.

Data of the patients were collected from patients’ records at the NCI, by direct contact with the patients at the outpatient clinic, and by phone calls to the patients themselves or their relatives.

*Study groups*

The study included thirty five patients with relapsed platinum sensitive epithelial ovarian cancer treated at the NCI between 2011 and 2017.These patients are divided into cases and controls:

- Cases: are fifteen in number who were treated with CRS and HIPEC with cisplatin 100 mg/m2 at temperature 41-42◦c for 60 minutes after receiving at least four cycles of conventional carboplatin and taxol. 

- Controls: are twenty in number who were treated with conventional chemotherapy carboplatin and taxol.

These patients had the following inclusion criteria; Patients aged between 20-70 years, with relapsed platinum sensitive epithelial ovarian cancer, whose recurrence was confined to the abdominal cavity, with no parenchymal liver deposits, with performance status (PS)≤ 2, (measured by the ECOG score).

Patients should have adequate respiratory, hepatic, cardiac, kidney function and bone marrow reserves, (normal chest X-rays or chest CT scans, (EF) 50% or more, normal liver enzymes, albumin level and serum creatinine. The adequacy of bone marrow reserves is defined as: HGB level 10mg /dl. or more,WBC 4,000 or more and platelet count not less than 150,000.

*Exclusion Criteria*

Non-epithelial or borderline ovarian tumor, pregnancy or breastfeeding, patients with severe impairment of respiratory, hepatic, renal function or inadequate bone marrow, patients with bowel obstruction or patients with secondary or tertiary recurrence or already submitted to HIPEC or those who have already made the second or third line chemotherapy.

*Pretreatment evaluation*

All patients had complete history and physical examination, complete blood count, liver and kidney function tests, coagulation profile, CA125 level, echocardiography, chest x-ray, computarized tomography of the abdomen and pelvis. CT chest and PET scan (optional, for selected cases).

The PCI is a scoring system to the intra abdominal carcinomatosis. This was confirmed at a consensus conference held in Milan in 2006 (Esquivel et al., 2007). The greatest possible extent of tumor involvement in each region is assessed using the lesion size score (from 0 to 3). The maximum possible number of points is thus 39, and the lowest 0.

The crucial anatomical sites are defined as anatomical sites where cancer becomes a systemic disease despite low PCI and at that point a palliative debulking without HIPEC is carried instead. These crucial anatomical sites where the liver parenchyma, the common bile duct, lymph nodes group involvement unrelated to the primary cancer and extensive mesenteric bowel involvement with the need for three or more resections. We adopted the completeness of cytoreduction score (CC), proposed by Sugarbaker: (CC-0 No residual tumor after cytoreduction; CC-1 < 2.5mm; CC-2 >2.5mm and <2.5cm; CC-3 residual nodules >2.5cm) (Sugarbaker, 2005). All cases in the study had CC score from 0-1.

*Grading systems of toxicity of chemotherapy*

In all cases and controls, the toxicity of chemotherapy was studied to compare the toxicity resulting from intraperitoneal chemotherapy versus toxicity from intravenous chemotherapy using WHO grading system.

*Statistical analysis*

Primary platinum free survival (PFS-1) was defined as the time elapsed between the end of primary treatment and first recurrence. The duration of secondary response (PFS-2) was defined as the time elapsed between HIPEC/other treatment (i.e. systemic chemotherapy) and secondary recurrence.The overall survival was defined as time at occurrence of the disease till date of death or date of last follow up. In this study PFS and OS were measured in months.

The cases and controls were followed up till the time of second recurrence, progression and death and censoring of patients was by documented death or date of last seen. 

Recorded data were analyzed using the statistical package for social sciences, version 20.0 (SPSS Inc., Chicago, Illinois, USA). Quantitative data were expressed as mean± standard deviation (SD). Qualitative data were expressed as frequency and percentage.

Kaplan-Meier method and Log rank test were used to estimate and to compare survival distribution between groups, the Independent-samples t-test of significance was used when comparing between two means, Chi-square (ϰ^2^) test of significance was used in order to compare proportions between two qualitative parameters. The confidence interval was set to 95% and the margin of error accepted was set to 5%. So, the p-value was considered significant if < 0.05, highly significant if <0.001 and p-value > 0.05 was considered insignificant.

## Results

This study is a retrospective case control study of 35 patients with recurrent platinum-sensitive epithelial ovarian cancer treated at the National Cancer Institute, Cairo University between 2011 and 2017.

The aim of the study is to compare CRS and HIPEC to systemic chemotherapy in patients with recurrent platinum-sensitive epithelial ovarian cancer with end point progression free survival and overall survival.

The two groups were homogenously matched, in terms of age, clinicopathological status, CA125 elevation, pretreatment parameters and PCI.

The PFS1 showed a median of 14 ±2.46 SE for cases (95% CI 9.18 and 18.82 respectively). The median PFS1for controls was 11±2.15 SE (95%CI 6.79 and 15.21 respectively). There was no statistically significant difference between the two groups (p-value 0.769).

*Intraoperative Parameters*

Two thirds (66.6%) of the patients had multiple disease while one third (33.3%) of the patients had diffuse disease. No one had single site recurrence. Number of surgical procedures performed was 16, as one patient had burst abdomen and was explored and had tension suture. All patients (100%) had abdominal and pelvic peritonectomy, while 60% had either supra or infracolic omentectomy. Diaphragmatic stripping was performed in 5 patients (33.3%), while splenectomy and cholecystectomy in 40% of the patients. Bowel resection was done in 6 patients (40%), 4(26.6%) had single resection and 2 (13.3%) had two resections. Lymph nodes involvement occurred in 4 patients (26.6%), of which 3 had pelvic nodes only, while one had pelvic and aortic lymph node involvement. Chest tube was inserted in 3(20%) of the patients. Iatrogenic injury occurred in 5 patients (33.3%) 3 had diaphragmatic tear, one had small bowel injury and one had urinary bladder tear (Table 1).

*Postoperative morbidity and mortality*

Two patients (13.3%) out from 15 died in the early post operative period (severe haemorrhage in one patient and DIC in the other). The most common complications were attributed to chemotherapy toxicity (53.3%), followed by pulmonary complications (33.3% needed mechanical ventilation, 13.3% had chest infection). Wound infection represented the third most common complication and occurred in 3 patients (20%). All patients needed blood and plasma transfusion while one patient needed cryoprecipitate and another patient needed platelets (Table 2).

The duration of ICU admission ranged from 1-23 days with a mean of 6.87±6.96 SD. The overall hospital stay ranged from 4-30 days, with a mean of 14.33±7.55 SD. Two patients (13.3%) needed readmission to the ICU The most common type of toxicity in cases was hepato-toxicity as four out of the eight (50%) events were liver toxicity. Neurotoxicity didn’t occur in cases. On the other hand neurotoxicity was the commonest type of toxicity in controls with nine events (39%) while no hepatic toxicity occurred in controls.

The percentage of each grade of toxicity in cases and controls were as follows: 8 (53.3%) patients of the cases experienced toxicity of chemotherapy of whom 4 (26.7%) had grade one toxicity, 2 (13.3%) had grade 2, 2 (13.3%) had grade 3 and 0% had grade 4 toxicity. On the other hand, all patients in controls had experienced toxicity of chemotherapy with 23 events in 20 patients 5 (25%), 10 (50%), 7 (35%) and 1 (5%) for grades 1,2,3 and 4 respectively. Statistically significant difference existed between the two groups in favor of the cases who received intraperitoneal chemotherapy versus the controls who received intravenous chemotherapy, with p-value 0.003 

*Survival Evaluation*

**Figure 1:**
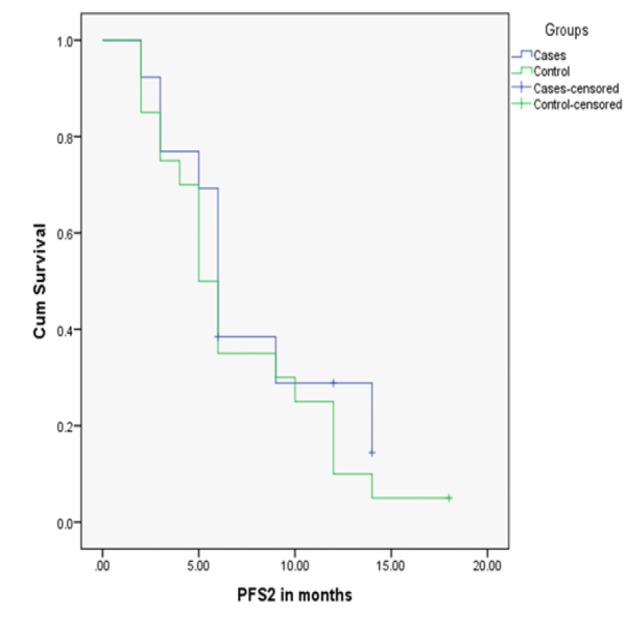
Comparison of PFS2 in Months in the Cases and Controls.

The PFS2 (Figure 1) was measured in cases and showed a median of 6 ±0.44SE, (CI 95%with 5.14-6.86 respectively), with a range of 2-14 months. The PFS2 was measured in controls with a median of 5±0.64 SE, (CI 95% with 3.75-6.25 respectively), with a range of 2-18. There was no statistically significant differences between cases and controls and p-value was 0.350.

**Figure 2:**
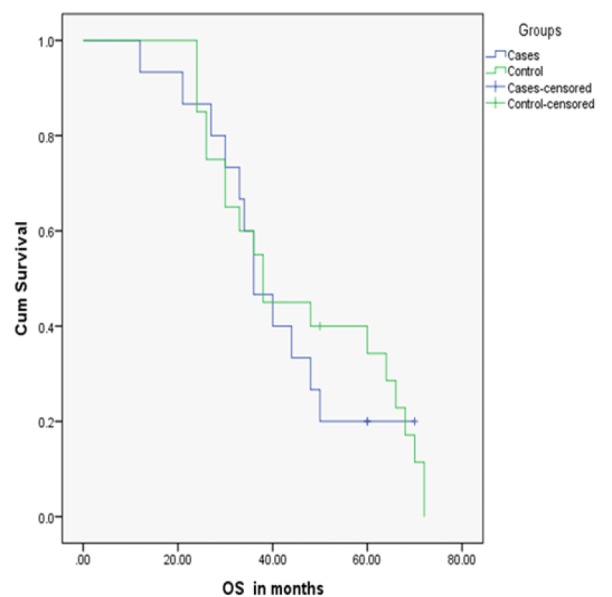
Comparison of OS in Cases and Controls

The OS (Figure 2) was measured in cases and controls with a median of 36 months in cases (range 21-70) and 38 months in controls (range 24-72). No statistically significant difference was found between cases and controls in OS as P-value was 0.711.

## Discussion

Epithelial ovarian cancer (EOC) causes more deaths than any other malignancy affecting the female reproductive system. In up to 75% of the patients, the disease is diagnosed at an advanced stage, with peritoneal involvement or distant metastasis (International Federation of Gynecology and Obstetrics (FIGO) stage III to IV) (Sun et al., 2016).

**Table 1:** The Intraoperative Parameters of the Cases

100%	15	No
Type of recurrence		
Single	0	0%
Multiple	10	66.60%
Diffuse	5	33.30%
Site of recurrence		
Lymph node involvement	4	26.60%
Aortic only	0	0%
Pelvic only	3	20%
Aortic/pelvic	1	6.60%
Intraparenchymal disease		
Splenic	2	13.30%
Hepatic	0	0%
Ascites	4	26.60%
No of surgical procedures performed	16	106.60%
Abdominal/pelvic peritonectomy	15	100%
Diaphragmatic stripping	5	33.30%
Aortic lymphadenectomy	1	6.60%
Pelvic lymphadenectomy	4	26.60%
Bowel resection	6	40%
Single	4	26.60%
Multiple	2	13.30%
Splenectomy	6	40%
Cholecystectomy	6	40%
Appendectomy	5	33.30%
Omentectomy	9	60%
Supracolic	7	46.60%
Infracolic	2	13.30%
Chest tube insertion	3 (bilateral)	20%
Iatrogenic injury	5	33.30%
Diaphragmatic tear	3	20%
Small bowel	1	6.60%
Urinary bladder	1	6.60%

**Table 2:** Mortality and Morbidity Events

No	15	100%
Tansfusion	15	100
Blood	15	100
Plasma	15	100
Others(platelets, cryoprecipitate)	2	13.30
30 days mortality	2	13.30
Post operative morbidity		
Haemorrhage	1	6.60
DIC	1	6.60
Pleural effusion (drained)	1	6.60
Pneumonia	0	0
Chest infection	2	13.30
ARDS	2	13.30
Heart arrhythmia	0	0
Heart failure	1	6.60
DVT/pulmonary embolus	0	0
Septicemia	0	0
Central venous line infection	1	6.60
Abdominal collection	1	6.60
Wound infection	3	20
Toxicity of chemotherapy	8	53.30
Bowel obstruction	0	0
Fistula	0	0
Burst abdomen	1	6.60
Need for mechanical ventilation	5	33.30

DIC, disseminated intravenous coagulopathy; DVT, deep venous thrombosis; ARDS, adult respiratory distress syndrome.

Unfortunately, the majority of patients, up to 70%, who present with advanced stages of disease, exhibit recurrent or persistent disease following primary treatment, despite extensive cytoreduction and six cycles of platinum based chemotherapy (Le Brun et al., 2014).

Optimal treatment of ovarian cancer has continued to be a vexing clinical dilemma, especially in the setting of recurrent disease. There has been no curative treatment for EOC peritoneal carcinomatosis. Repeated conventional chemotherapy alone or in combination with molecular targeting agents could improve survival and quality of life at the cost of considerable treatment-related adverse events (Yoshida et al., 2015).

Bristow et al., (2009) published a recent meta-analysis of nonrandomized cohorts of patients treated with secondary surgery for an ovarian cancer relapse. Authors showed that the proportion of patients with complete cytoreductive surgery was independently associated with the best overall postrecurrence survival rate. As in front-line treatment, the surgical objective of secondary cytoreduction should be the resection of all macroscopic disease with no gross residual. 

Relapse appears mainly as a peritoneal carcinomatosis. Taking into account this intraperitoneal (IP) pathway of widespread, intraperitoneal chemotherapy is an obvious option of treatment to consider. In a recent feasibility study of IP chemotherapy for ovarian cancer relapse, authors stated on its feasibility, with a high completion rate and encouraging survival rates, with a mean overall survival of 51 months (Skaznik-Wikiel et al., 2012).

This study is the experience of the NCI in the recurrent ovarian cancer. Strict criteria for cases and controls selection was achieved. The age was between 20 and 70 years. The PFS1 was 6 months or more to insure platinum senistivity. Patients with parenchymal liver diseases were excluded, together with patients with lymph node metastases beyond the groups of the primary tumor. 

The completion of cytoreduction was achieved to CC0-CC1. In cases, patients who needed more than two bowel resections were also excluded. The PCI was calculated for all cases using conventional imaging. All cases had PCI ≤ 20 preoperatively, however, on laparotomy 9 patients out of 15 were upgraded and of which 6 reached a score more than 20. Sugarbaker suggested that favorable outcomes were achieved with PCI ≤ 20 (Sugarbaker, 2005). However, in our study this cut off point of PCI score at 20 or less was not adopted, and any score more than 20 was included provided CC0-1 was achieved.

The mean PCI of cases was 18.20±6.96 ranging from 6-27. Similar to our study many studies with variable designs did not adopt a PCI 20 or less in inclusion criteria. Sun et al., in his study included 46 patients with median PCI of 20 (7-39) (Sun et al., 2016). Barkin et al., (2012) in his study revealed PCI ranging from 1-30 in a total of 246 patients of relapsed ovarian cancer. Deraco et al., (2012) in his multi-institutional study, reported a PCI 15.2 (4-30).

The twenty patients of controls were carefully matched to the cases in order to minimize bias and to draw careful conclusions of the efficacy of the treatment. No statistical significant differences between the two groups in age, clinicopathological aspects, CA125 elevation, pretreatment parameters, PS and PFS1.

The PCI was calculated in cases and controls. There was a statistically significant difference between cases and controls in favor of the cases (p-value 0.048). This meant that the cases had higher PCI score that may affect the homogenousity of the two groups. However, the PCI of cases was calculated after the surgery with maximal degree of accuracy in comparison to controls in which PCI depended on radiological findings (CT scan and PET/CT). As the controls were never explored there was no way to accurately calculate the PCI as in cases.

In clinical staging of the PCI, the accuracy, specifity and sensitivity of the procedure appear different in the current scientific literature depending on the size of the peritoneal lesions. For lesions larger than 5 cm, the specificity of the CT scan in all the abdominal regions is 100% with a sensitivity of 94%. For lesions smaller than 5cm, sensitivity ranges from 88% with a specificity of 60% to a sensitivity of 11%. The CT has a lower sensitivity in the small intestine (8%-17%) and it is 11% for lesions larger than 5 mm and smaller than 5cm (Suidan et al., 2014). Consequently, the clinical PCI is significantly underestimated for small lesion and miliary dissemination, with an incidence of false-negatives for the small intestine and mesentery of 35% (Level 1 evidence) (Rivard et al., 2014). PET/ CT reaches up to 70% sensitivity and up to 100%preoperative specificity for stage 2 and 3 disease but under values lesions of nodules smaller than 5mm in all quadrants (level 2 evidence) (Rubini et al., 2014).

These level 1 and 2 evidence regarding the underestimation of the conventional radiology for the PCI scores were replicated in this study. By calculating the PCI of cases before surgery based on radiology and after surgery, the PCI was upgraded in 9 (60%) out of 15 cases and under graded in 1 patient. The calculated preoperative PCI in cases was compared to that of controls with no statistically significant difference between the two groups (p-value 0.156).

All patients were followed for at least 24 months. The median follow up period was 36 in cases and 38 in controls. All patients relapsed during the follow up period.

The PFS2 revealed a median of 6 months (range 2-14) in cases and 5 months (range 2-18) in controls. The median OS was 36 and 38 months in cases and controls respectively. No statistically significant difference between the cases and controls were observed in PFS2 and OS (P-value, 0.350 and 0.711 respectively). However, the PFS2 was in favor of cases and OS was in favor of controls without reaching significance. The percentage of patients who survived 5 years or more was 20% in cases and 35% in controls. 

Faggotti et al., (2012) matched thirty patients with recurrent platinum sensitive ovarian cancer underwent CRS and HIPEC, Oxaliplatin (OXA) 460 mg/m^2^ at the temperature of 41.5°C (cases) to thirty seven patients who had either systemic chemotherapy or CRS without HIPEC. The median PFS1 was 20 and 22 months for cases and controls respectively. After at least 24 months of follow up, The median duration of secondary response (PFI-2) was 26 months in the Cases (range 5–73) with respect to 15 months (range 4–58) in the Controls (p=0.004). Moreover, the Cases survived longer than the Controls, with a 2 and 5 year overall survival after treatment of 96.7% and 68.4% vs. 68.4% and 42.7% months, respectively (p=0.017). 

These differences between Faggotti’ s study and our study is attributed to more selection criteria in the former study, ( more patients had single site recurrent, no patients had ascites, PS was 0-1).

In the 1st Evidence-based Italian consensus conference on CRS and HIPEC for peritoneal carcinomatosis from ovarian cancer which was held in 2015 and published in 2017, the panel adopted the DESKTOP scores for prediction of completion of cytoreduction (PS 0-1, absence of residual tumor from the primary surgery and absence of ascites). This tool score predicts complete cytoreduction in 76% of the patients undergoing cytoreductive surgery (Cavaliere et al., 2017). The Faggotti’s study adopted 2 criteria of the DESKTOP score (PS 0-1 and absence of ascites). This may explain the different of results between the two studies.

In an 8-year period, Spiliotis et al.,randomized 120 women with advanced ovarian cancer (FIGO IIIc and IV) who experienced disease recurrence into two groups. Group A comprised 60 patients treated with CRS followed by HIPEC and then systemic chemotherapy. Group B comprised 60 patients treated with CRS only and systemic chemotherapy.

The mean survival for group A was 26.7 versus 13.4 months in group B (p-value 0.006). Three-year survival was 75 % for group A versus 18 % for group B (p-value 0.01).

In the HIPEC group, the mean survival was not different between patients with platinum-resistant disease versus platinum-sensitive disease (26.6 vs. 26.8 months). On the other hand, in the non-HIPEC group, there was a statistically significant difference between platinum-sensitive versus platinum-resistant disease (15.2 vs. 10.2 months, p-value 0.002). Complete cytoreduction was associated with PCI score of 15 or less appeared also to have longer survival. The authors concluded that the use of HIPEC along with the extent of the disease and the extent of cytoreduction play an important role in the survival of patients with recurrence in an initially advanced ovarian cancer (Spiliotis et al., 2015).

In Spiliotis ‘ study, there was a statistically significant difference in OS of both groups in favor of the HIPEC group which was failed to be proven in our study. However the mean OS in cases and controls were 26.7 and 13.4 respectively, while in our study the median were 36 and 38 respectively. This can be explained by the fact that Spiliotis ‘ study included stage 3,4 patients with platinum sensitive and platinum resistant recurrence.

In the 1st Evidence-based Italian consensus conference mentioned before the panel declared that the rate of complications reported in maximal CRS with HIPEC in primitive or relapse OC are varied ranged between 0% and 65.2% (Cavaliere et al., 2017) and these data are level 1 evidence. The major complications in our study reached 46.5% matching the rate mentioned in this consensus.

The most common surgery related complications included anastomotic leakage, intestinal perforation, intraperitoneal hemorrhage and abdominal evisceration (Cavaliere et al., 2017). In our study no patient had anastomotic leakage, one patient had iatrogenic small intestinal tear (6.6%), one had intraabdominal hemorrhage (6.6%), and one had abdominal evisceration (6.6%).

A recent study published in the British Journal Of Cancer has proven that during the 90 minutes of HIPEC, the hematic dosage toxicity threshold is never exceeded, even when the dual intraperitoneal drug is administered (cisplatin and paclitaxel) (Ansaloni et al., 2015). These data are replicated in our study as only one case (6.6%) experienced hematological toxicity thrombocytopenia, and it was grade I toxicity which meant that the platelet level ranged from 75,000-99,000.

Regarding the overall toxicity from chemotherapy there was significant difference between cases and controls in the rate of chemotherapeutic toxicity in favor of cases. In cases 53.3% had toxicity versus 100% in controls.(p-value 0.003).

In a recent review focused on the last ten years of scientific literature and refered to 143 publications involving 1450 patients with ovarian cancer treated with HIPEC, a total of 493 of them received hyperthermic chemotherapy as a primary treatment while 957 received it following a relapse. Among this, 499 patients from 8 studies had homogenous clinical characteristics and represented the most homogenous population, on which conclusions can be drawn with a higher level of interest. In this population,the median overall survival was 37.3 months (range 27-78), the median disease free survival is 14.4 months (range 12-30), and the median 5 year OS 40% (range 28-71) (Chiva and Gonzalez-Martin, 2015).

In our study the median OS was 36months, the median disease free survival was 6 months and 5years OS was 20%. The Italian consensus mentioned before adopted this review considering it level 2 evidence (Cavaliere et al., 2017).

Limitations of this study were in the retrospective nonrandomized design of the trial and in the limited number of patients, but the close matching between cases and controls should theoretically assure the quality of data. 

In conclusion, according to our study, CRS and HIPEC does not seem to have impact on OS and PFS in the the setting of recurrent platinum sensitive ovarian cancer. The only issue in favor of HIPEC is the significant reduction in chemotherapeutic toxicity when given by the intraperitoneal way (p-0.003). 

As the selection criteria of the patients candidate for CRS and HIPEC are evolving, we recommend on going researches with much more refined selection criteria and with larger sample size.
